# GFR measurement in patients with CKD: Performance and feasibility of simplified iohexol plasma clearance techniques

**DOI:** 10.1371/journal.pone.0306935

**Published:** 2024-07-17

**Authors:** Fabiola Carrara, Flavio Gaspari, Matias Trillini, Tobia Peracchi, Diego Fidone, Nadia Stucchi, Silvia Ferrari, Daniela Cugini, Norberto Perico, Aneliya Parvanova, Giuseppe Remuzzi, Piero Ruggenenti

**Affiliations:** 1 Istituto di Ricerche Farmacologiche Mario Negri IRCCS, Clinical Research Center for Rare Diseases “Aldo e Cele Daccò”, Bergamo, Italy; 2 Unit of Nephrology, Azienda Socio-Sanitaria Territoriale (ASST) Papa Giovanni XXIII, Bergamo, Italy; University of Sao Paulo Medical School, BRAZIL

## Abstract

Implementing shortened one-compartment iohexol plasma clearance models for GFR measurement is crucial since the gold standard inulin renal clearance technique and the reference two-compartment, 10-hour, 16-samplings iohexol plasma clearance method are clinically unfeasible. Inulin may precipitate anaphylactic shock. Four-hour and 8-hour one-compartment iohexol plasma clearance models with Bröchner-Mortensen correction provide accurate GFR measurements in patients with estimated GFR (eGFR) > or ≤40 mL/min/1.73m^2^, respectively. We compared the performance of the simplified 5-hour, 4-samplings, two-compartment population pharmacokinetic model (popPK) with the performance of the reference two-compartment 10-hour iohexol method in 16 patients with GFR 15.2 to 56.5 mL/min/1.73 m^2^. We also compared the performance of shortened (5, 6 and 7-hour) one-compartment models with the performance of the standard 8-hour one-compartment model in 101 patients with eGFR ≤40 mL/min/1.73 m^2^. The performance of popPK and shortened methods versus reference methods was evaluated by total deviation index (TDI), concordance correlation coefficient (CCC) and coverage probability (CP). TDI <10%, CCC ≥0.9 and CP >90% indicated adequate performance. TDI, CCC and CP of popPK were 11.11%, 0.809 and 54.10%, respectively. All shortened, one-compartment models overestimated the GFR (p <0.0001 for all) as compared to the 8-hour model. TDI, CCC and CP were 7.02%, 0.815, and 75.80% for the 7-hour model, 7.26%, 0.803, and 74.20% for the 6-hour model, and 8.85%, 0.729 and 64.70% for the 5-hour model. The agreement of popPK model was comparable to that obtained with the Chronic-Kidney-Disease-Collaboration-Epidemiology (CKD-Epi) and the Modification-of-Diet-in-Renal-Disease (MDRD) serum-creatinine based equations for GFR estimation. PopPK model is remarkably unreliable for GFR measurement in stage III-IV CKD patients. In patients with eGFR ≤40 mL/min/1.73m^2^, shortened one-compartment models, in particular the 5-hour model, are less performant than the reference 8-hour model. For accurate GFR measurements, the iohexol plasma clearance should be measured with appropriate protocols. Over-simplified procedures should be avoided.

## Introduction

The plasma clearance of unlabeled iohexol has been introduced to directly, safely and accurately measure the GFR. This procedure allows avoiding the use of radioactive tracers and of renal clearance procedures that are difficult to apply in clinics and research. Notably, the inulin renal clearance technique, that is considered the gold-standard procedure for GFR measurement–is not allowed any longer because of evidence that exposure to inulin can precipitate serious hypersensitivity reactions, including anaphylactic shock [1–4]. Among different plasma clearance protocols, the 10-hour, 16-samplings, two-compartment plasma clearance protocol has emerged as the reference method for GFR measurement [5,6]. However, it is excessively time- and cost-consuming and virtually unfeasible in everyday clinical practice.

**Fig 1:**
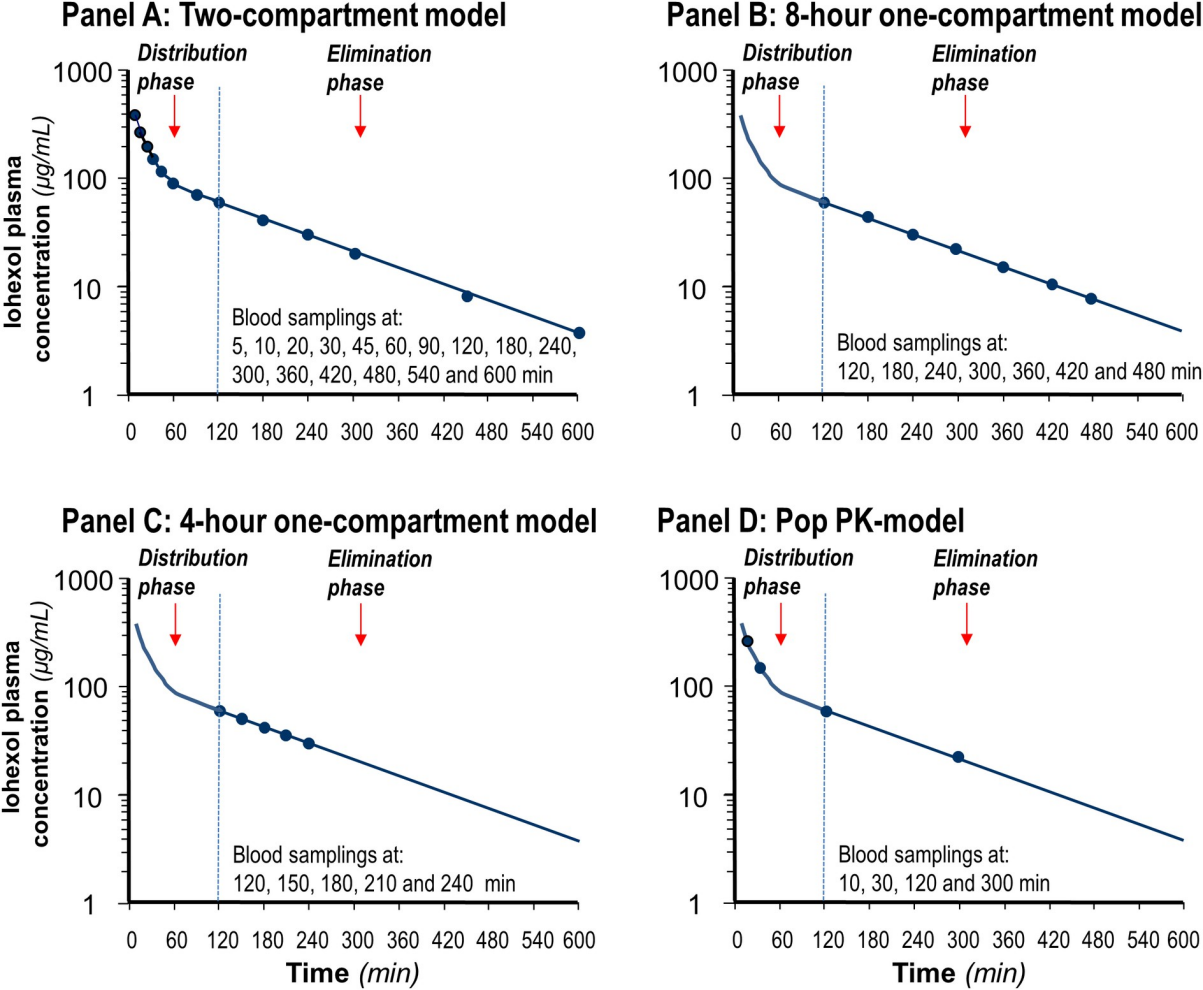
Blood sampling scheme. Blood samplings required for the standard 4-hour and 8-hour one-compartment models (Panels C and B), for the reference 10-hour two-compartment model (Panel A), and the simplified two-compartment popPK model (Panel D).

We previously found that in patients with eGFR >40 mL/min/1.73 m^2^, the GFR can be accurately measured by using a 4-hour one-compartment iohexol plasma clearance model requiring five blood samples collected from 120 to 240 minutes after injection of the tracer, whereas in those with eGFR ≤40 mL/min/1.73 m^2^ the GFR can be accurately measured by an 8-hour one-compartment model with seven blood samples collected from 120 to 480 minutes after injection (Fig 1, Panels C and B) [7,8]. Notably, the performance of these two simplified, one-compartment models was validated versus the gold-standard 10-hour, two compartment model (Fig 1, Panel A) [7,8]. The reproducibility of GFR data obtained with the two (4-hour and 8-hour) one-compartment models was confirmed by studies comparing results of serial GFR measurements performed in a cohort of patients with different levels of kidney dysfunction, including those with near-terminal kidney failure [8]. Then, the good performance of both methods was confirmed in several clinical trials [9–16] and cohort studies [17].

However, whereas the 4-hour protocol can be easily used in patients with eGFR >40 mL/min/1.73 m^2^, even in everyday clinical practice, the 8-hour protocol implemented for patients with eGFR ≤40 mL/min/1.73 m^2^ is admittedly excessively time-consuming. This may limit its use in clinics and research. To facilitate GFR measurements in patients with eGFR ≤40 mL/min/1.73 m^2^, further shortened methods have been tested but, again, with discouraging results [18–23]. Indeed, the use of methods based on an excessively reduced number of blood samples, increases the impact of analytical errors, even in the assay of one single sample. Thus, one single error in the measurement of iohexol plasma concentration can compromise the reliability and reproducibility of the whole plasma clearance procedure for GFR measurement.

The simplified, two-compartment iohexol population pharmacokinetic model (popPK) based just on four timed blood samples collected at 10 and 30 minutes after injection during the iohexol distribution phase and at 120 and 300 minutes during the iohexol elimination phase (Fig 1, Panel D), has been recently reported to overcome the aforementioned drawbacks and to provide reliable GFR measurements for patients with GFR either >40 and ≤40 mL/min/1.73 m^2^ [24]. Thus, we tested the performance of this additional simplified two-compartment model versus the reference 10-hour, 16-samplings, two-compartment iohexol plasma kinetic model.

Moreover, in order to implement simplified methods in particular in patients with eGFR ≤40 mL/min/1.73 m^2^ we compared the performance of shortened one-compartment methods with that of the reference 8-hour method.

On the other hand, we considered that no additional studies were needed to implement further simplified clearance models for patients with eGFR >40 mL/min/1.73 m^2^ since the currently available 4-hour one-compartment model is accurate and, at the same time, easily applicable in clinics as well as in research.

## Materials and methods

### GFR measurement

All GFR measurements were centralized at the Laboratory of the Clinical Research Center for Rare Diseases “Aldo and Cele Daccò” of the Istituto di Ricerche Farmacologiche Mario Negri IRCCS, in Bergamo (Italy). Between June 1991 and July 1993, GFRs were measured by using the standard 10-hour, 16-samplings, two-compartment iohexol plasma clearance technique [7]. The performance of this method was confirmed by analyses comparing GFR values obtained with the measurement of iohexol plasma clearance with those obtained by measuring the renal clearance of inulin in patients with a wide range of GFR values [7].

Then we implemented two simplified, one-compartment models corrected with the Bröchner-Mortensen formula [25], for GFR measurement in patients with eGFR > or ≤40 mL/min/1.73 m^2^, and validated these simplified procedures by analyses comparing the GFR values obtained with these two simplified models with the GFR values obtained with the reference two-compartment model and even with the inulin renal clearance technique [7]. The precision of these two methods has been determined by evaluating the reproducibility of GFR values obtained with repeated measurements in three different occasions during a 12-day period in 24 patients with renal disease and a creatinine clearance ranging from 14 to 104 mL/min /1.73 m^2^.

Both simplified methods were based on the measurement of iohexol plasma concentration in blood samples collected during the elimination phase of the tracer, whereas no sample was collected during the distribution phase. For patients with eGFR >40 mL/min/1.73 m^2^ we validated a 4-hour protocol with five blood samples collected at 120, 150, 180, 210 and 240 minutes after iohexol injection, whereas for patients with eGFR ≤40 mL/min/1.73 m^2^ we validated an 8-hour protocol with seven blood samples collected at 120, 180, 240, 300, 360, 420, 480 minutes after the injection (Fig 1, Panels C and B). [25].

The aforementioned clearance procedures were used in all patients referred to the Clinical Research Center of the Istituto di Ricerche Farmacologiche Mario Negri IRCCS who required a direct GFR measurement for clinical or research purposes and had no history of allergy to radiocontrast agents. All patients provided written informed consent to the procedures and to the use of their clinical laboratory data. According to the March 31, 2008 determination of the Agenzia Italiana del Farmaco (https://www.gazzettaufficiale.it/eli/id/2008/03/31/08A02109/sg), no specific patient informed consent and Ethical Committee approval was required because of the retrospective, purely observational nature of the study that was based on already available data–that had been already used for previous studies [9,10,26]—and did not require any additional clinical or laboratory data from considered patients.

### Laboratory evaluations

All laboratory evaluations were centralized at a single laboratory at the Clinical Research Center. Iohexol plasma levels were measured by high-performance-liquid chromatography (HPLC) as previously described [27]. GFR values were normalized to 1.73 m^2^ of body surface area (BSA) calculated with DuBois & DuBois formula [28]. The morning of each iohexol clearance study, serum creatinine concentration was measured with the modified rate Jaffe´ traceable to isotope dilution mass spectrometry (IDMS) using an automatic device (Beckman Synchron CX5, Beckman Coulter S.p.A., Cassina De’ Pecchi, Italy) and demographic and anthropometric data considered in CKD-Epi and MDRD equations [29,30] were recorded.

### Phase A study

In this phase we tested the performance of the simplified two-compartment popPK model (Fig 1, Panel D) by using GFR data obtained with 40 clearance studies performed in 16 patients [median (range) number of clearances per patient: 3 (1–5)] with the standard 10-hour, 16-samples, two-compartment model (Fig 1, Panel A).

We measured GFR with pop PK model by using published web tool available at http://folk.uio.no/anderas/iohexol.html [24]. We also compared the performance of the popPK model with that of CKD-Epi and MDRD equations.

### Phase B study

In this phase we tested the performance of 7, 6 and 5-hour one-compartment models by using GFR data obtained with 216 standard 8-hour clearance studies [median (range) number of clearances per patient: 2 (1–7)] performed between July 2017 and June 2022 in 101 patients with eGFR ≤40 mL/min/1.73 m^2^ (Fig 1, Panel B). Again, we compared the performance of the shortened one-compartment models with that of CKD-Epi and MDRD equations.

### Statistical analysis

GFR values calculated with the standard two-compartment and the 8-hour one-compartment model were compared with GFR values obtained with the simplified two-compartment popPK and 7, 6 or 5-hour one-compartment models, respectively, by paired or unpaired t-test as appropriate. Correlations between variables were evaluated by Pearson r correlation coefficient and analyzed using the ANOVA test followed by the Student Newman-Keuls post-hoc analysis. Data were expressed as mean ± SD, median and interquartile range (IQR) or numbers and percentages as appropriate. The statistical significance level was defined as P <0.05.

Mean percent error (P10, P15) was the percentage of each simplified GFR value within 10 and 15% of the standard GFR value. Taking into account that the reproducibility of the iohexol plasma clearance is 6.28% [8], the GFR values lying within the 10% error range were *a priori* considered as virtually identical to the GFR values obtained with the reference method. The agreement between GFR measurements obtained by standard reference methods or simplified procedures was assessed by the limits of agreement described by Bland-Altman [31] and by specific statistics for continuous data, including evaluation of the concordance correlation coefficient (CCC), total deviation index (TDI) and coverage probability (CP) as proposed by Lin et al. [32,33]. The limits of agreement are a simple graphic tool which describes the limits that include the majority of the differences between two measurements. The narrower these limits are, the better the agreement. The CCC combines elements of accuracy and precision. Its scores range from 0 to 1 and a value >0.9 reflects optimal concordance between measurements [32]. TDI is a measure that captures a large proportion of data within a boundary for allowed differences between 2 measurements [33]. CP is the probability that the differences between methods lie within the boundary of a tolerance interval of 5 mL/min/1.73 m^2^ [34]. The ideal situation is to have a TDI <10%, meaning that 90% of the estimations fall within an error of ±10% from the reference method. This is based on previous reports on the reproducibility of measured GFR considering different methods [35]. Finally, these statistics provide confidence intervals that allow generalization of results.

Bland-Altman plots show the relationship between the difference between target and observed measurements and the mean of both [31]. Bias was defined as the mean value of the difference between each GFR value obtained by standard or simplified methods. Confidence intervals for bias were calculated by means of bootstrap methods [36–38]. A Mountain plot was created by computing a percentile for each ranked difference between the GFR obtained by standard and simplified measurement methods [39]. In order to take into account repeated measures per participant, the intercept random model using PROC MIXED from SAS 9.4 software was used.

The estimates of the agreement index were calculated using ’lme4’ function from ’lmer’ package, their 95% confidence intervals were established by exploiting bootstrap tecnique using R 4.1[36–38].

## Results

Baseline characteristics including different parameters of kidney function of patients included in Phase A and Phase B of the study are shown in Table 1. Overall, study patients had stage III-IV CKD. There was a predominance of male patients and, as expected, patients with lower GFRs tended to be older than patients with higher GFRs.

**Table 1:** Baseline characteristics and kidney function parameters of study participants included in Phase A and Phase B.

	Phase A	Phase B
**Patients** *(n)*	16	101
**Age** *(years)*	51.8 ± 15.5	59.6 ± 12.4
**Weight** *(kg)*	74.6 ± 12.0	71.1 ± 12.8
**Height** *(cm)*	173.4 ± 9.5	167.8 ± 9.6
**BMI** *(kg/m*^*2*^*)*	25.7 ± 4.2	25.2 ± 3.8
**Male sex, n** (%)	14 (87%)	57 (56%)
**Serum Creatinine** *(mg/dL)*	2.1 ± 0.6	2.2 ± 0.8
**eGFR with CKD-Epi** *(mL/min/1*.*73 m*^*2*^*)*	36.2 ± 10.6	31.8 ± 11.0*
**eGFR with MDRD** *(mL/min/1*.*73 m*^*2*^*)*	36.7 ± 10.5	30.7 ± 10.0*
**mGFR with reference two-compartment model** *(mL/min/1*.*73 m*^*2*^*)*	38.2 ± 9.8	-
**mGFR with reference 8-hour one-compartment model** *(mL/min/1*.*73 m*^*2*^*)*	-	28.6 ± 6.5
**mGFR with popPK model** *(mL/min/1*.*73 m*^*2*^*)*	40.6 ± 11.9	-
**mGFR with 7-hour one-compartment model** *(mL/min/1*.*73 m*^*2*^*)*	-	28.9 ± 6.6*
**mGFR with 6-hour one-compartment model** *(mL/min/1*.*73 m*^*2*^*)*	-	29.3 ± 6.8*
**mGFR with 5-hour one-compartment model** *(mL/min/1*.*73 m*^*2*^*)*	-	29.9 ± 6.8*

Data are mean±SD or number and percentage

*P<0.05 paired sample t-test vs. reference model. popPK = pharmacokinetic population model.

### Phase A study: Comparisons between standard and popPK two-compartment models

**Fig 2:**
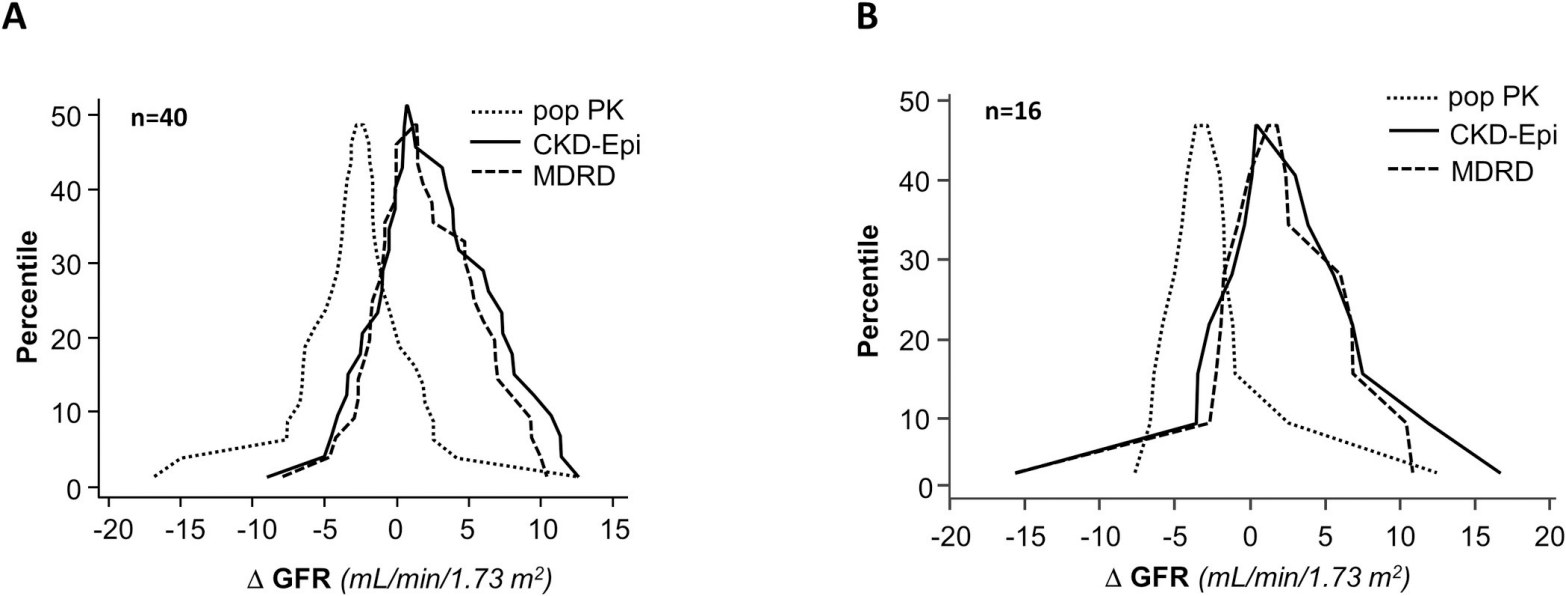
Mountain plot analysis. Mountain plot analysis showing a percentile for each ranked difference between the standard 10-hour two-compartment model (reference GFR) and the simplified two-compartment popPK model (popPK GFR), MDRD (MDRD eGFR) and CKD-Epi (CKD-Epi eGFR) in the study group considered as a whole (Panel A, Overall, n = 40) and only first, baseline clearance studies available from each individual patient (Panel B, Baseline Analyses, n = 16).

**Fig 3:**
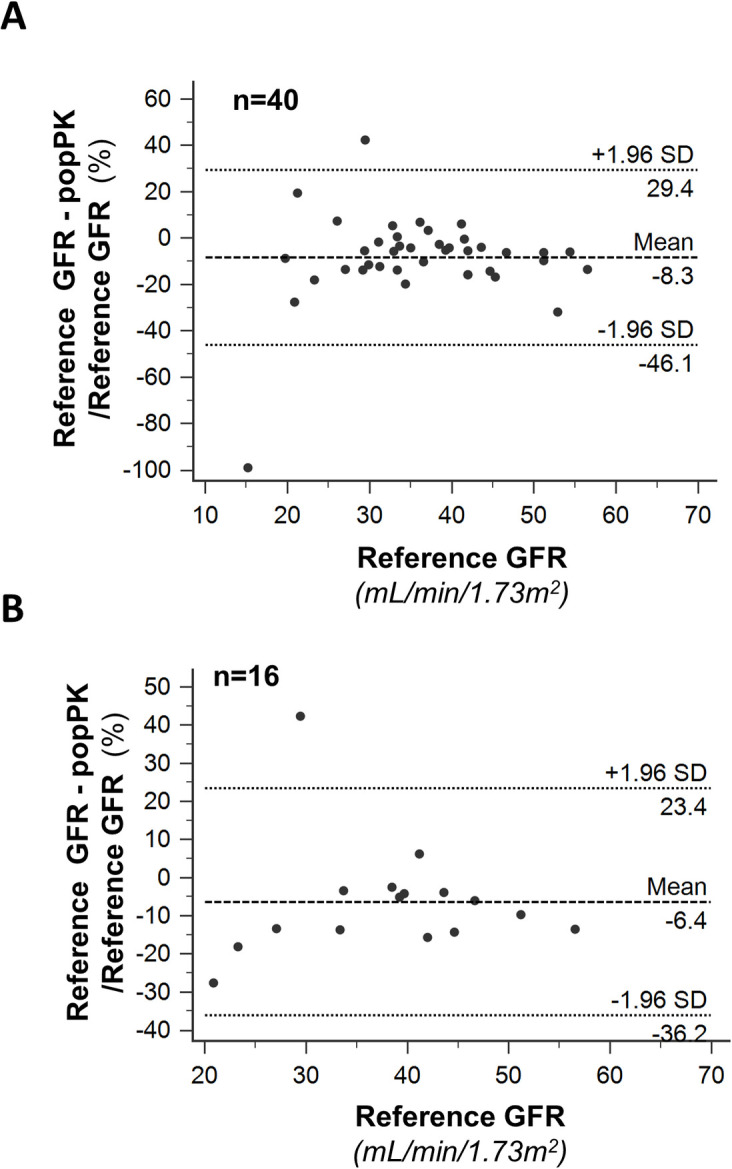
Bland–Altman plots obtained with popPK model and the standard 10-hour two-compartment model. Bland–Altman plots and limits of agreement between GFR obtained with the simplified two-compartment popPK model (popPK GFR) and the standard 10-hour two-compartment model (reference-GFR) in the study group considered as a whole (Panel A, Overall, n = 40) and only first, baseline clearance studies available from each individual patient (Panel B, Baseline Analyses, n = 16).

**Fig 4:**
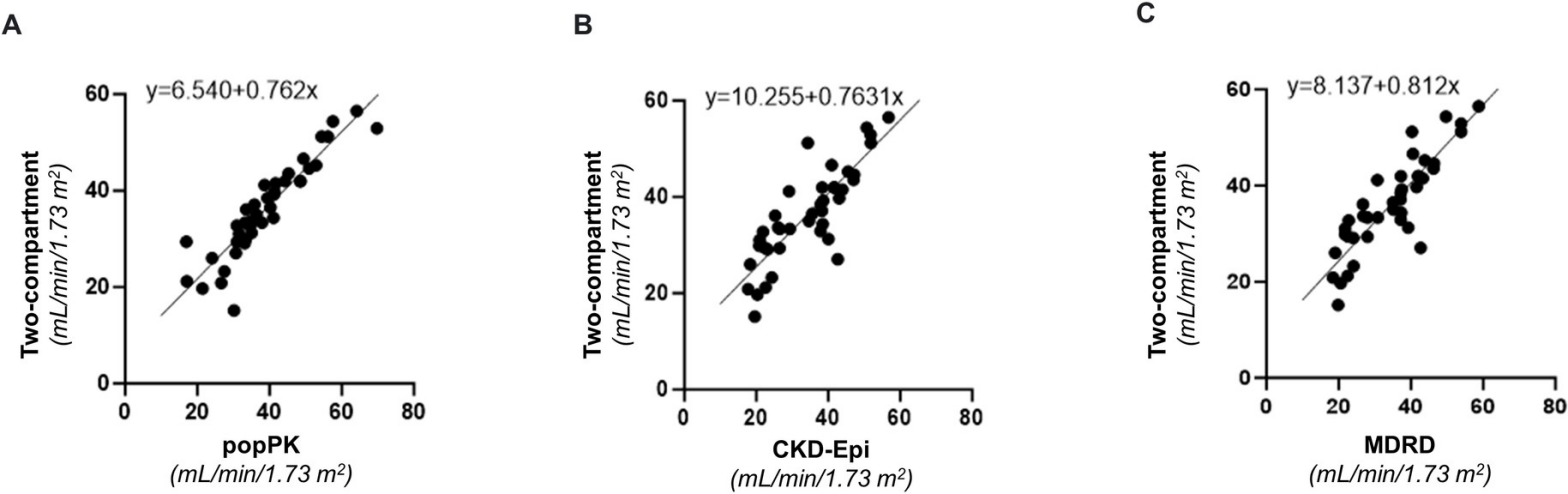
Intercept random model for Phase A. Intercept random model based on standard 10-hour two-compartment model (reference GFR) versus the simplified two-compartment popPK model (popPK GFR), MDRD (MDRD eGFR) and CKD-Epi (CKD-Epi eGFR) (n = 40).

GFR values obtained with forty 10-hour, 16-point, two-compartment clearance studies performed in 16 patients with chronic kidney disease were compared with the GFR values obtained in the same patients during the same clearance procedures by using the popPK two-compartment model. GFR values obtained with the standard two-compartment model averaged 36.3±9.8 mL/min/1.73 m^2^ and ranged from 15.2 to 56.5 mL/min/1.73 m^2^ whereas GFR values obtained with the popPK model averaged 39.1±11.9 mL/min/1.73 m^2^ and ranged from 17.0 to 69.8 mL/min/1.73 m^2^ (S1 Table). PopPK model slightly overestimated GFR (Fig 2, Panel A). P10 was 55% and TDI averaged 11%, a percentage that indicates that 90% of the popPK estimations had an error ranging from—11 to + 11%, CCC averaged 0.809, a value that reflected good precision and accuracy. TDI values obtained with the popPK model (11%) were comparable to those obtained with CKD-Epi (10%) and MDRD (9%) equations (Table 2). Finally, CP averaged 54%, a value that indicated that the error of 60% of the estimations exceeded ± 5 mL/min/1.73 m^2^. The Mountain plots showed that the distribution curve of the popPK model deviated to the left from “0” point, a finding consistent with systematic GFR overestimation (Fig 2, Panel A). Moreover, the wide distribution of GFR data in the popPK plot was consistent with wide random error of the simplified versus the standard two-compartment method (Fig 2, Panel A). Consistently, the Bland–Altman plot comparing GFR values obtained with the popPK model and the standard 10-hour, two-compartment model showed wide limits of agreement that ranged from -46.1 to 29.4% (Fig 3, Panel A). Similar findings were obtained considering only baseline clearance studies available for each individual patient (Fig 2, Panel B and Fig 3, Panel B). The mixed linear regression analyses for popPK model showed slopes of 0.762 (IC 95% 0.651 to 0.872) and intercepts of 6.540 (IC 95% 1.896 to 11.183), MDRD showed slopes of 0.812 (IC 95% 0.566 to 1.058) and intercepts of 8.137 (IC 95% -1.324 to 17.598) and CKD-Epi showed slopes of 0.7631 (IC 95% 0.493 to 1.034) and intercepts of 10.255 (IC 95% -0.087 to 20.597). The overall performance of popPK was not superior to that of CKD-Epi and MDRD equations (Table 2 and Fig 4).

**Table 2:** Agreement of the Performance of the simplified two-compartment popPK model, CKD-Epi and MDRD versus the standard 10 hour two compartment model in 40 clearance studies performed in 16 patients considered as a whole (Overall).

	popPK	CKD-Epi	MDRD
**CCC**	0.809 (0.668 to 0.866)	0.806 (0.575 to 0.883)	0.839 (0.664 to 0.896)
**CP**	54.1(47.00 to 69.30)	57.2 (49.60 to 63.80)	61.50 (53.80 to 68.0)
**TDI**	11.11* (8.04 to 13.10)	10.40 (9.02 to 12.30)	9.47 (8.27 to 11.19)
**P10**	55	50	55
**P15**	75	60	65

TDI total deviation index, CCC concordance correlation coefficient, CP coverage probability (95% CI bootstrap)

*wide TDI (goal within 10%).

### Phase B study: Comparisons between standard 8-hour and shortened 7, 6 and 5-hour one-compartment models

**Fig 5:**
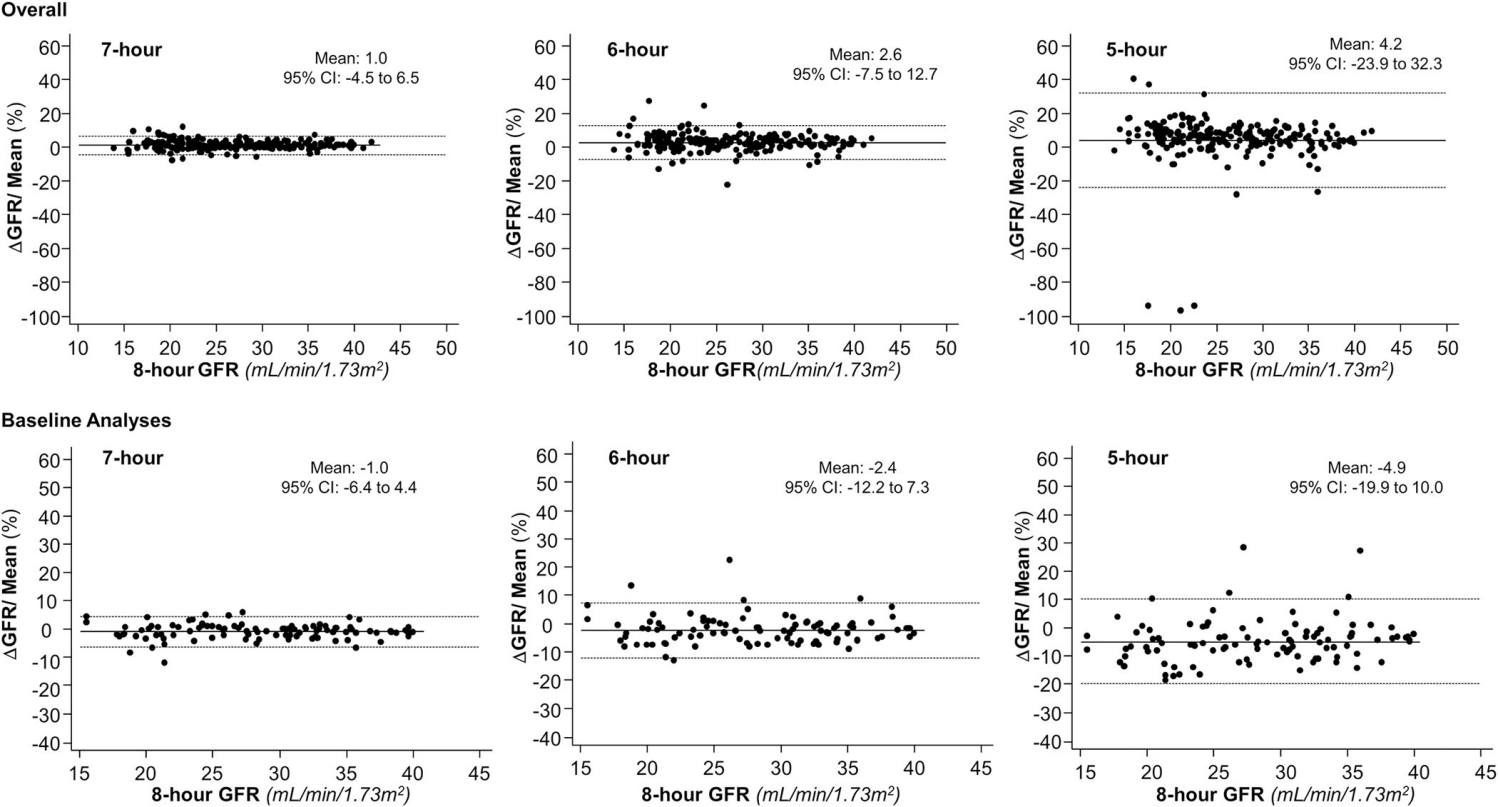
Bland–Altman plots with 8-hour vs. 7, 6 and 5 hours after iohexol infusion. Bland–Altman plots and limits of agreement between GFR values obtained with the one-compartment models shortened to 7, 6, or 5 hours versus the GFR values obtained with the standard 8-hour procedure. Upper panels show data obtained from all available clearance measurements (Overall); Lower panels show data obtained from the first, baseline, clearance measurements available from each individual patient (Baseline Analyses).

**Fig 6:**
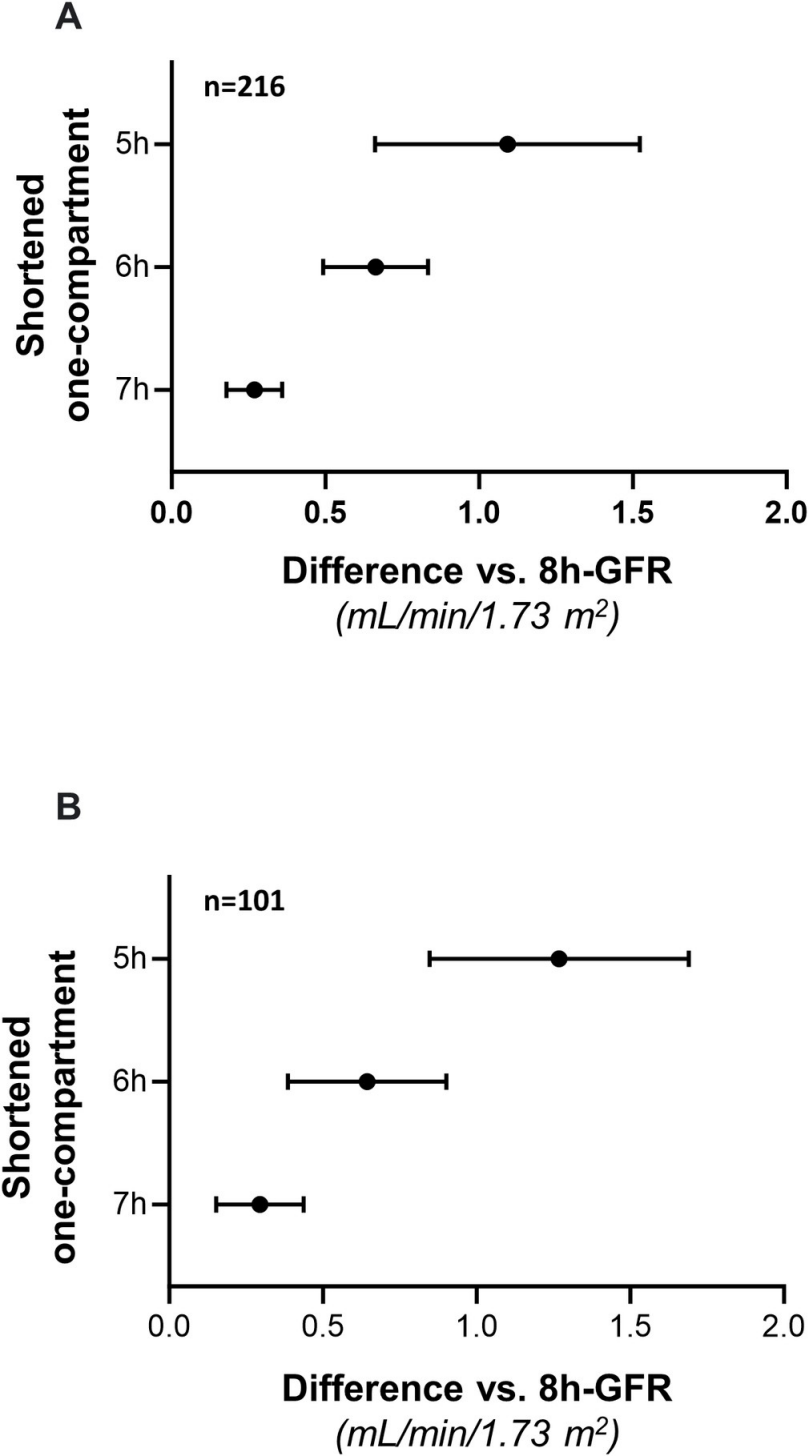
GFRs measured with 8-hour vs. 7, 6 and 5 hours after iohexol infusion. GFR values measured with the 8-hour standard one-compartment model versus GFR values obtained with shortened one-compartment protocols stopped at 7, 6 and 5-hours after iohexol infusion considering all available clearance studies (Panel A, Overall, n = 216) and only first, baseline clearance studies available from each individual patient (Panel B, Baseline Analyses, n = 101). Data are mean ± 95% confidence intervals.

**Fig 7:**
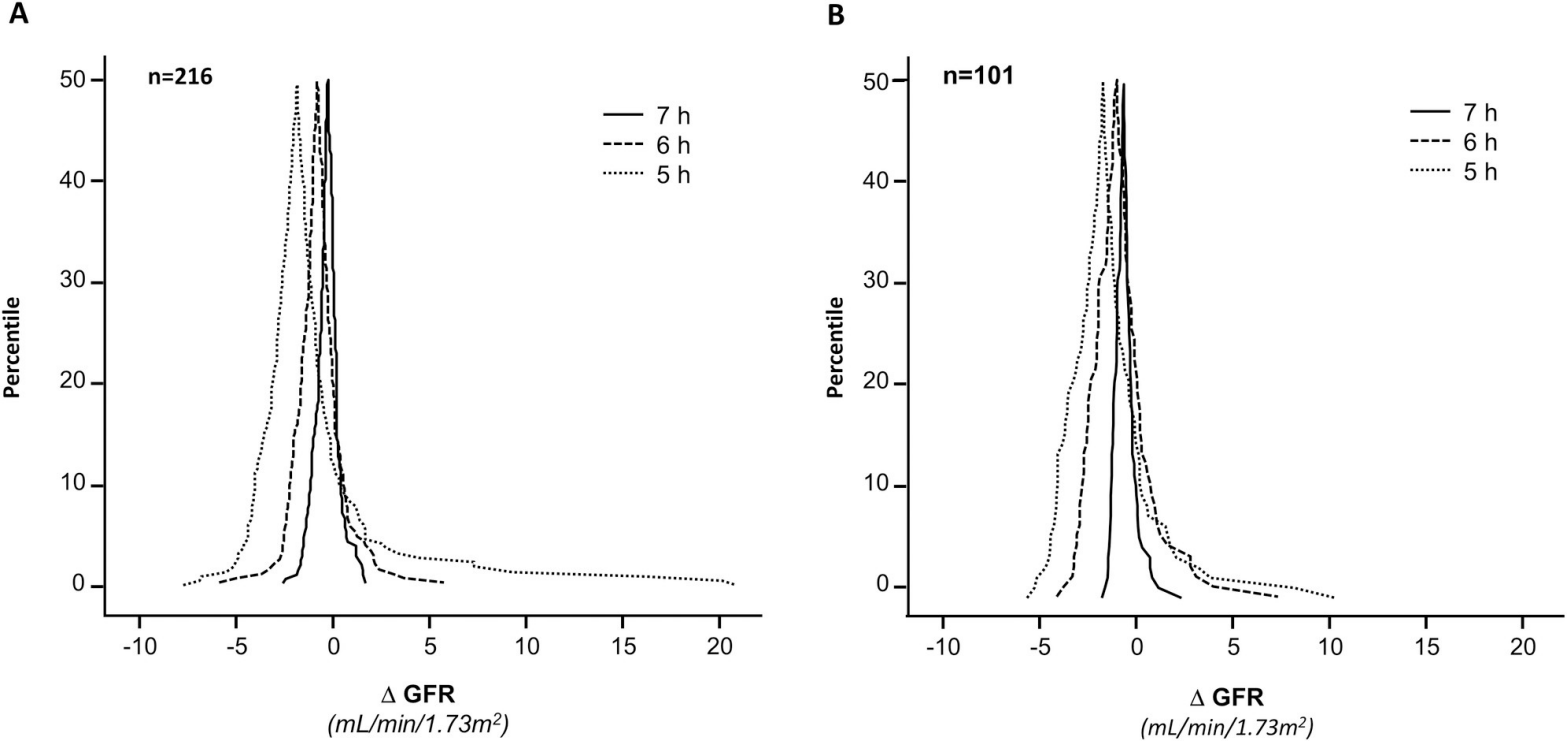
Mountain plot with 8-hour vs. 7, 6 and 5 hours after iohexol infusion. Mountain plot analysis showing a percentile for each ranked difference between the standard 8-hour one-compartment model (8-hour GFR) and different shortened protocols (7, 6 and 5-hours GFR) and Plot analysis was performed by using GFR data obtained from all available clearance measurements (Panel A, Overall, n = 216) and only from the first, baseline, clearance measurement available from each individual patient (Panel B, Baseline Analyses, n = 101).

Two-hundred-sixteen GFR values obtained by 8-hour one-compartment iohexol kinetic profiles of 101 patients with eGFR ≤40 mL/min/1.73 m^2^ were compared with the GFR values obtained during the same clearance procedures by using the shortened 7-, 6- and 5-hour one-compartment models. GFR values obtained with the standard 8-hour model averaged 26.6±6.6 mL/min/1.73 m^2^ and ranged from 13.9 to 42.0 mL/min/1.73 m^2^, whereas GFR values averaged 26.8±6.7 mL/min/1.73 m^2^ and ranged from 13.8 to 43.0 mL/min/1.73 m^2^ with the 7-hour model, averaged 27.2±6.8 mL/min/1.73 m^2^ and ranged from 13.7 to 44.1 mL/min/1.73 m^2^ with the 6-hour model and averaged 27.7±7.6 mL/min/1.73 m^2^ and ranged from 0.6 to 46.0 mL/min/1.73 m^2^ with the 5-hour model. The Upper Right Panel of Fig 5. clearly shows that there are three unreliable GFR values. Thus, GFR overestimation increased in parallel with shortening of the clearance procedure (Fig 6). All GFRs obtained with the shortened models exceeded the GFR value obtained with the reference 8-hour one-compartment model (Fig 6). Consistently, the Mountain plots showed that the distribution curves of shortened models deviated to the left from the ‘‘0” point. This finding confirmed the systematic GFR overestimation, in particular with the 5-hour model. Again, the degree of the error increased in parallel with the shortening of the procedure, and the largest error was observed with the 5-hour model (Fig 7, Panel A). The Bland–Altman plots comparing GFR values obtained with different shortened models with those obtained with the standard 8-hour method showed narrow limits of agreement (from—4.5 to 6.5%) with the 7-hour model (Fig 5, Upper Panel, Overall, n = 216).

**Fig 8:**
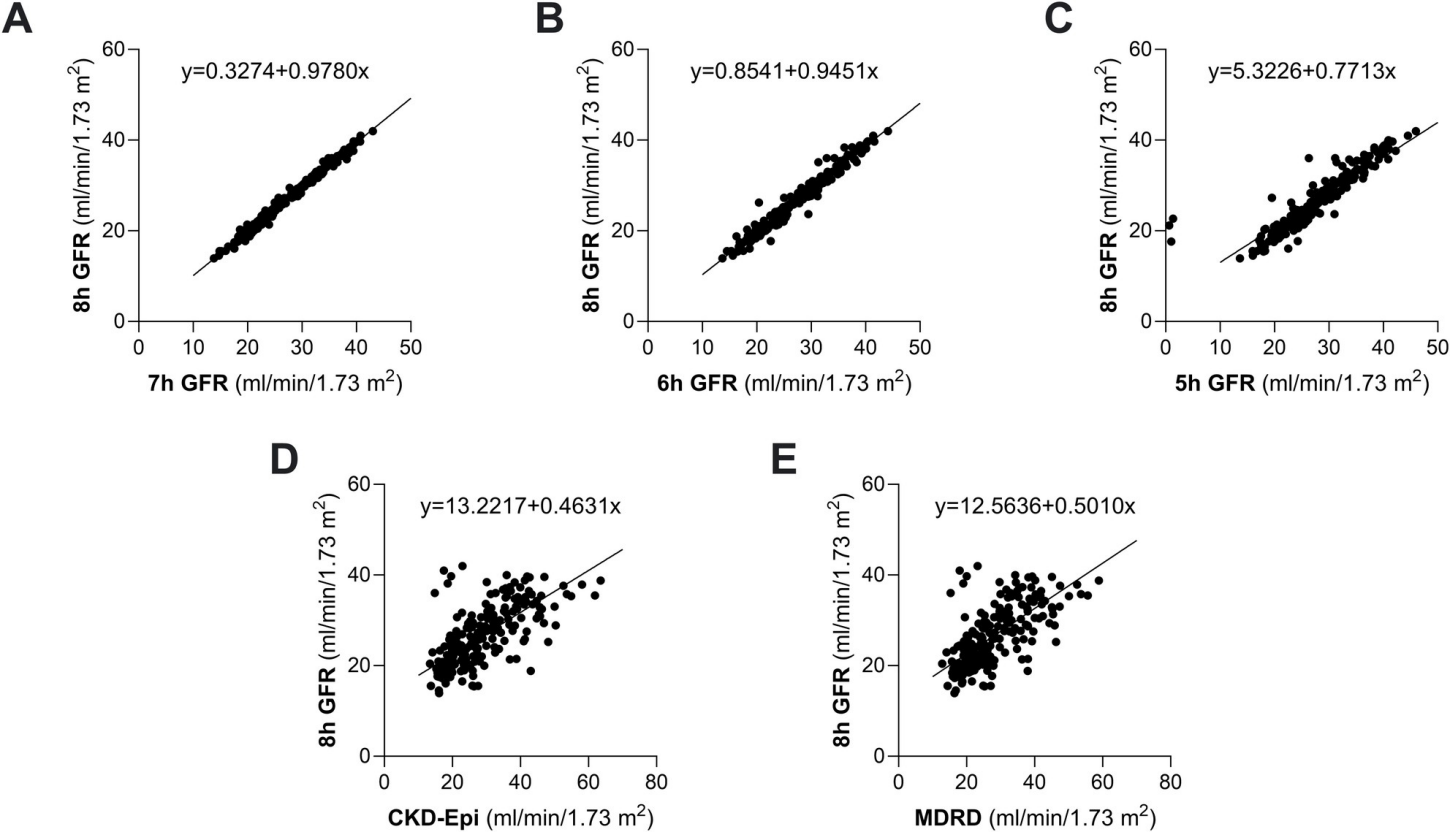
Intercept random model for Phase B. Intercept random model based on GFR values measured with the 8-hour standard one-compartment model versus GFR values obtained with 7, 6 and 5-hours models and CKD-Epi and MDRD formulas (n = 216).

These limits widened (– 7.5 to 12.7%) with the 6-hour model and widening was even larger (-23.9 to 32.3%) with the 5-hour model (Fig 5, Upper panels Overall, n = 216). Percentage error of shortened protocols as compared to the reference 8-hour model, ranged from -8.09 to 11.87% with the 7-hour model, from -22.3 to 27.4% with the 6-hour model and from -97.0 to 40.3% with the 5-hour model. TDI and CP were adequate for all shortened models. However, when compared to 7- and 6-hour models, the 5-hour model had a worse performance. The CP of the 5-hour model CP averaged 65%, a value that indicated that 35% of the estimations had an error that exceeded ± 10% (Table 3). Similar findings were obtained considering only baseline clearance data (Figs 6B and 7, Panel B). The mixed linear regression analyses showed that the 7- and 6-hour one-compartment models perform similarly to the 8-hour model, whereas the performance of the 5-hour model and of serum creatinine based equations was inferior to that of the 8-hour model (Fig 8).

**Table 3:** Performance of shortened 7-, 6- and 5-hour one compartment models and of CKD-Epi and MDRD equations versus the standard 8-hour one-compartment model in 216 clearance studies performed in 101 patients with e GFR <40 ml/min/1.73 m^2^.

	7-hour	6-hour	5-hour	CKD-Epi	MDRD
**CCC**	0.815 (0.736 to 0.876)	0.803 (0.725 to 0.865)	0.729 (0.651 to 0.785)	0.631 (0.517 to 0.704)	0.664 (0.541 to 0.733)
**CP**	75.8 (67.8 to 84.3)	74.2 (66.6 to 82.5)	64.7 (58.7 to 72.1)	48.3 (42.0 to 55.0)	52.6 (45.9 to 59.4)
**TDI**	7.02 (5.81 to 8.30)	7.26 (6.07 to 8.50)	8.85 (7.59 to 10.05)	12.68* (10.89 to 14.84)	11.47* (9.89 to 13.46)
**P10**	99	94	70	32	38
**P15**	100	98	90	51	54

TDI total deviation index, CCC concordance correlation coefficient, CP coverage probability (95% CI bootstrap)

*wide TDI (goal within 10%).

## Discussion

GFR is widely accepted as the best overall index of kidney function in health and disease. GFR decline is correlated with decline in other functions of the kidney, such as tubular reabsorption and secretion, and metabolic and endocrine function. Decreased GFR is associated with complications of acute and chronic kidney disease. GFR level is also the criterion to define the stage of chronic kidney disease and is the ideal marker to monitor the efficacy of treatments aimed to delay disease progression in patients with kidney dysfunction. Moreover, GFR reduction is probably the strongest predictor of increased cardiovascular morbidity and mortality [40]. Thus, accurate and reliable GFR measurements are crucial in clinics and research.

Calculation of the plasma clearance of non-radiolabeled exogenous markers such as iothalamate and iohexol by using a standardized two-compartment model is a reliable and safe method for GFR measurement that has been validated by studies comparing GFR values obtained with adequate plasma clearance models with those obtained with the inulin renal clearance technique, the gold-standard method for GFR measurement [7,41–43]. However, this two-compartment model needs sixteen serial blood samples collected over a period of ten hours during the distribution and elimination phases of the infused tracer, which limits its use in clinics and research. Thus, we implemented two simplified one-compartment models that, with the Bröchner-Mortensen correction, generate GFR values with an accuracy and precision comparable to those of the reference two-compartment model, without the evaluation of the distribution phase of the tracer. Both models were validated versus the renal inulin clearance technique, although in a limited number of small-sized studies [7,8]. Both models, however, are time-consuming and demanding for physicians, nurses and patients and are difficult to apply in every-day clinical practice. Thus, implementing further simplified and shortened plasma clearance procedures to increase feasibility without affect data accuracy, would have major implications in clinics and research.

On this regard, in Phase A of our present two-phases study we showed that the recently proposed simplified, two-compartment population pharmacokinetic model (popPK) [24] poorly performed as compared to the standard 10-hour, two-compartment model in GFR measurement in patients with CKD Stage III-IV. The use of the popPK to replace the use of CKD-Epi or MDRD is not justified because the performance of popPK and of the two equations is similarly poor and the popPK model is obviously much more expensive and resource-demanding. This finding could be explained by the fact that popPK model is based on just two sampling points for each kinetic phase (the distribution and the elimination phase, respectively: Fig 1). Consequently, even a slight error in one of these measurements during one of the two phases could affect the reliability of the entire procedure. Conversely, the availability of a higher number of sampling points that are fitted by a nonlinear regression program according to a one-compartment model [42], allows to ‘smooth’ the impact of possible random analytical errors in one single plasma sample. This results in a better fitting and thus in a robust and accurate GFR determination.

In Phase B we demonstrated that in subjects with eGFR ≤40 mL/min/1.73 m^2^, 7-, 6- and 5-hour one-compartment models generated GFR values that appeared to sufficiently agree with those obtained with the reference 8-hour one-compartment model. Thus, in theory, these shortened models could increase the feasibility of GFR measurement, in particular in patients requiring close renal function monitoring, and reduce costs, improve patient compliance, and facilitate screening programs without introducing major errors. However, the 5-hour model was associated with a worsened TDI (although this finding was probably acceptable considering the a priori established 10% TDI).

In conclusion, shorter GFR protocols offer advantages such as convenience, cost-effectiveness, reduced burden on healthcare facilities, and allow more frequent monitoring of renal function. However, over-simplified clearance procedures such as the two-compartment popPK model and the 5-hour one compartment model do not perform adequately and are not recommended. Indeed, results of Phase A and Phase B analyses showed that the performance of the popPK model and the 5-hour one-compartment models was comparable to that of serum creatinine-based CKD-Epi and MDRD equations. Moreover, in some patients the 5-hour one-compartment model generated really unreliable GFR values.

Our present findings may help optimizing currently available procedures for the diagnosis of kidney dysfunction and renal disease monitoring and treatment in clinics and research. Indeed, a GFR <60 mL/min/1.73 m^2^ is taken to define CKD and a GFR <20 mL/min/1.73 m^2^ can be an indication for considering patient inclusion in a waiting list for a kidney transplantation or placement of a vascular or peritoneal access for extravascular or peritoneal chronic dialysis, respectively [44]. Accurate GFR measurement is also crucial to modulate the posology of drugs with a predominant renal clearance, in particular for those with a narrow therapeutic window [45] and its regular monitoring might help designing trajectories of GFR decline over time in order to precisely monitor the effect of nephro-protective interventions aimed to delay worsening kidney function or prevent progression to ESKD.

## Limitations and strengths

The major limitation of the present study is the relatively small number of study participants. However, this limitation is at least in part mitigated by the large number of available clearance procedures, up to five per patient in Phase A and nine per patient in Phase B. Moreover, also the iohexol plasma clearance technique has limitations related to the fact that iohexol has a high protein binding that may limit its ultrafiltration and is minimally reabsorbed at tubular level [46]. However, protein binding is reported to be <2% [47,48]. This may result in a slight GFR underestimation. On the other hand, the remarkably good mean intra-individual coefficient of variation (5.59%) and reproducibility index (6.28%) confirmed the accuracy and reliability of the reference 8-hour one-compartment model used in repeated measurements in subjects with pre-terminal kidney failure [8]. Moreover, the performances of the different clearance models were compared by using the same iohexol concentration measurements performed in plasma samples obtained during the same identical clearance studies in the same individuals. This approach, reduced any possible intra-individual data variability, which increased the power of the analyses. Study results can be generalized to the large majority of adult patients with CKD as they were obtained from study participants with kidney dysfunction ranging from CKD stage III to CKD stage IV. This is the patient population that is most often in need of accurate GFR measurements to monitor/predict progression of renal disease, test the response to nephroprotective or even nephrotoxic medications and modulate the posology of drugs with renal clearance and/or narrow therapeutic windows. No data in pediatric patients were available.

The major strength is the accuracy in the conduction of the clearance procedures that were all performed at a single, dedicated Clinical Research Center and the measurements of the iohexol plasma concentrations that were all centralized in the same Laboratory [23,49]. In addition, the ten-hour, two-compartment and the 8-hour one-compartment models were previously validated by comparing their performance with that of the renal clearance of inulin, the “gold standard” for GFR measurement [7]. However, in the present study we could not directly validate the performance of simplified iohexol plasma clearance procedures versus the inulin renal clearance technique because inulin is no longer allowed for human use because of major safety concerns related to the excess risk of serious hypersensitivity reactions, including anaphylactic shock, reported upon exposure to the tracer [3]. Moreover, the reliability of inulin as an ideal marker of glomerular filtration has been challenged by evidence of some degree of extra-renal clearance of the molecule resulting in GFR overestimation [50].

## Conclusions

The recently proposed two-compartment popPK model does not provide any advantage over the 8-hour or the 4-hour standard one-compartment procedures to measure the GFR in CKD patients with Stage III-IV CKD and should be abandoned because it provides results that are not superior to those obtained with serum-creatinine based GFR estimation equations. On the other hand, the performance of the popPK model in less advanced CKD may merit further investigation.

For patients with eGFR ≤40 mL/min/1.73 m2, the 7- and 6- hour one-compartment models appear to provide sufficiently accurate and reproducible GFR values, although they tend to be over-estimated as compared to the GFR values obtained with the reference 8-hour, one-compartment clearance protocol. The 5-hour protocol is poorly performant and should be avoided. Thus, when accurate GFR measurements are needed in clinics and research, renal plasma clearance of unlabeled tracers such as iohexol should be measured with an appropriate protocol. Over-simplified procedures should be avoided.

## Supporting information

S1 Table Main characteristics and kidney function parameters of study participants included in Phase A and Phase B. (DOCX)

S2 Table Phase A study. (XLSX)

S3 Table Phase B study. (XLSX)
