# Supplementary material for: GFR measurement in patients with CKD: Performance and feasibility of simplified iohexol plasma clearance techniques
Source: PLoS One. 2024 Jul 17;19(7):e0306935. doi: 10.1371/journal.pone.0306935 (PMC11253958; doi:10.1371/journal.pone.0306935)
Supplement: S1 Table — (DOCX) [file pone.0306935.s001.docx]

|  | **Phase A** | | | | | **Phase B** | | |  |
| --- | --- | --- | --- | --- | --- | --- | --- | --- | --- |
|  |  | |  |  | |  |  |  |  |
| **Clearance studies** *(n)* | | 40 | | | 216 | | | | |
| **Clearances per patient** *- median (range)* | | 3 (1-5) | | | 2 (1-7) | | | | |
| **eGFR with CKD-Epi** *(mL/min/1.73 m^2^)* | | 34.3 ± 10.9 | | | 28.9 ± 10.3 | | | | |
| **eGFR with MDRD** *(mL/min/1.73 m^2^)* | | 34.7 ± 10.7 | | | 28.0 ± 9.4 | | | | |
| **mGFR with reference two-compartment model** *(mL/min/1.73 m^2^)* | | 36.3 ± 9.8 | | | - | | | | |
| **mGFR with reference 8-hour one-compartment model** *(mL/min/1.73 m^2^)* | | - | | | 26.6 ± 6.65 | | | | |
| **mGFR with popPK model** *(mL/min/1.73 m^2^)* | | 39.1 ± 11.9 | | | - | | | | |
| **mGFR with 7-hour one-compartment model** *(mL/min/1.73 m^2^)* | | - | | | 26.8 ± 6.7 | | | | |
| **mGFR with 6-hour one-compartment model** *(mL/min/1.73 m^2^)* | | - | | | 27.2 ± 6.8 | | | | |
| **mGFR with 5-hour one-compartment model** *(mL/min/1.73 m^2^)* | | - | | | 27.7 ± 7.6 | | | | |
| Data are mean±SD, median (IQR) or number. popPK= pharmacokinetic population model | | | | | | | | | |
|  | |  | | |  | | | | |
|  | |  | | |  | | | | |
|  | |  | | |  | | | | |
|  | |  | | |  | | | | |

**Table S1. Main characteristics and kidney function parameters of study participants included in Phase A and Phase B.**
